# Forgetting to take HIV antiretroviral therapy: a qualitative exploration of medication adherence in the third decade of the HIV epidemic in the United States

**DOI:** 10.1080/17290376.2021.1989021

**Published:** 2021-10-15

**Authors:** R. Freeman, M. Gwadz, K. Francis, E. Hoffeld

**Affiliations:** a Independent Consultant, Brooklyn, NY, USA; b New York University Silver School of Social Work, New York, NY, USA; c Centre for Drug Use and HIV Research (CDUHR), New York University School of Global Public Health, New York, NY, USA

**Keywords:** Qualitative, HIV antiretroviral therapy, adherence, forgetting, racial/ethnic disparities, HIV survivorship research

## Abstract

Optimal adherence to HIV antiretroviral therapy (ART) is challenging, and racial/ethnic disparities in adherence rates are substantial. The most common reason persons living with HIV (PLWH) give for missed ART doses is forgetting. We took a qualitative exploratory approach to describe, from the perspectives of African American/Black and Hispanic/Latino PLWH, what it means to forget to take ART and factors that influence forgetting. Participants (*N* = 18) were purposively sampled for maximum variability and engaged in semi-structured/in-depth interviews on HIV/ART management. The analysis took a directed content analysis approach. Participants were mostly male (56%) and African American/Black (79%), between 50 and 69 years old, and had lived with HIV for an average of 21 years. Findings were organised into six inter-related themes: (1) forgetting to take ART was a shorthand description of a complex phenomenon, but rarely a simple lapse of memory; (2) ‘forgetting’ was means of managing negative emotions associated with HIV; (3) life events triggered mental health distress/substance use which disrupted adherence; (4) historical traumatic events (including AZT monotherapy) and recent trauma/loss contributed to forgetting; (5) patient-provider interactions could support or impede adherence; and (6) intrinsic motivation was fundamental. Implications for HIV social service and health care settings are described.

## Introduction

The challenges inherent in taking medication daily for a chronic and stigmatised condition such as HIV infection cannot be overstated (Vervoort, Borleffs, Hoepelman, & Grypdonck, 2007). Yet, in the United States over the past three decades, the field of HIV has seen great progress in the effectiveness and tolerability of antiretroviral therapy (ART), along with increasing numbers of persons living with HIV (PLWH) taking ART with optimal adherence and thereby achieving and sustaining HIV viral suppression (Centers for Disease Control and Prevention, 2017). This, in turn, has resulted in improved quality of life and greater longevity for a substantial proportion of the population of PLWH (Trickey et al., 2017). Indeed, with proper resources and supports in place, many PLWH experience HIV as a manageable, chronic condition (Deeks, Lewin, & Havlir, 2013; Ernst, 2017; Jelliman & Porcellato, 2017).

Despite these advances, however, serious gaps along the HIV care continuum persist. Of the estimated 1.1 million individuals living with HIV in the United States, 63% have received HIV care, but only approximately half are retained in continuous HIV care (49%) and/or evidence HIV viral suppression (51%) (Centers for Disease Control and Prevention, 2018). Moreover, there are significant racial/ethnic disparities in HIV incidence, prevalence, and health outcomes (Beer, Mattson, Bradley, & Skarbinski, 2016; Samji et al., 2013). For example, compared to White PLWH, African American/Black and Hispanic/Latino PLWH evidence longer times between diagnosis with HIV and ART initiation, and between ART initiation and achieving HIV virologic suppression (Haines et al., 2016). Further, even after controlling for socio-demographic factors, depression, and substance use, African American/Black PLWH evidence lower average ART adherence rates compared to White PLWH (Simoni et al., 2012), and African American/Black and Hispanic/Latino PLWH are less likely to sustain HIV viral suppression compared to White PLWH (Crepaz, Dong, Wang, Hernandez, & Hall, 2018). Past quantitative research has documented the complex factors that underlie adherence challenges, including structural-, socioeconomic-, health care system-, treatment-, condition-, and patient-related factors, which disproportionately affect African American/Black and Hispanic/Latino populations from low socio-economic status backgrounds (Rebeiro et al., 2017; Vervoort et al., 2007).

Consistent adherence to ART is critical for achieving sustained HIV viral suppression. Ideally, PLWH will take at least 95% of ART doses on schedule, although approximately 80% adherence rates may be acceptable for achieving HIV viral suppression for the newer ART regimens (Byrd et al., 2019). (For the present study, the term ‘optimal adherence’ will refer to 80–100% of ART doses taken as prescribed.) Since the earliest days of the HIV pandemic, forgetting to take ART doses has been the primary reason PLWH provide to explain suboptimal adherence patterns (Barfod, Sørensen, Nielsen, Rodkjær, & Obel, 2006; Chesney et al., 2000; Harzke et al., 2004; Sauceda, Neilands, Johnson, & Saberi, 2018). In response, the early literature initially called for adherence interventions to prevent forgetting that emphasised health education, problem-solving skills, and practical tips, along with external memory aids such as reminders, pillboxes, visual aids, calendars, and more recently, text message reminders and smartphone apps (Amico, Harman, & Johnson, 2006; Mayer & Fontelo, 2017). Yet this approach, which focuses mainly on individual behaviour and that is primarily psychoeducational and cognitive in nature, has shown insufficient success in improving ART adherence rates (Kalichman et al., 2015; Magidson, Blashill, Safren, & Wagner, 2015).

More recently, forgetting to take ART has been linked with psychosocial factors such as substance use issues, depression, disruption of daily routines, medical distrust, and mental health issues (Kalichman, Kalichman, & Cherry, 2017; Vervoort et al., 2007; Wilson & Childs, 2002). Other studies have drawn linkages between forgetting to take ART doses and structural factors associated with low socioeconomic status (Saberi et al., 2015) as well as stigma and disclosure concerns (Wilson & Childs, 2002). These lines of research have suggested that to support PLWH in sustaining high levels of ART adherence, approaches that take into consideration and/or address these contributing factors are needed, in addition to interventions that seek to improve adherence-related behavioural skills (Saberi et al., 2015). Yet, in the context of a typical short healthcare or social service encounter, providers may have no choice but to take patients at face value when they state that forgetting is the main reason for missed ART doses (Harzke et al., 2004). Thus, these short health care encounters may represent missed opportunities for patients to articulate the range of factors that underlie or cause forgetting to take ART, and further, the short encounters also impede patients from being able to generate solutions to suboptimal ART adherence.

While forgetting to take one’s ART medication has been the most commonly reported barrier to ART adherence throughout the epidemic, empirical evidence suggests that forgetting may stand in for other, less easily articulated or less socially desirable reasons for missing doses of ART (Barfod et al., 2006; Dicicco-Bloom & Crabtree, 2006; Sauceda et al., 2018). As Harzke et al. (2004) note, although numerous previous studies have identified forgetting as a significant self-reported barrier to ART adherence, less is known about the specific contexts within which forgetting occurs, or the factors most closely associated with forgetting to take ART.

The present study seeks to extend this past research on understanding barriers to optimal ART adherence and takes a qualitative and descriptive approach to explore the most common reason PLWH give for why they miss ART doses; namely, forgetting, from the perspectives of African American/Black and Hispanic/Latino PLWH and in the context of the third decade of the epidemic. We focus in particular on individuals with past experiences of sustained optimal adherence to ART along with periods of suboptimal adherence (e.g. missing several doses a week, or taking less than half of prescribed ART doses over substantial periods), to uncover the structural, social, and individual-level factors that influence ART adherence, including individual decision-making about what it means to be optimally adherent. Guided by the theory of triadic influence (Flay, Snyder, & Petraitis, 2009), a multi-level social-cognitive theory, we sought to uncover and explore how a range of factors; namely, those at individual-, social-, and structural-levels of influence, along with potential historical and cultural influences, were intertwined with and simultaneously presented within the theme of forgetting to take ART. Thus, rather than seeing forgetting as an independent, isolated barrier to consistent ART adherence, the present study sought to understand forgetting as imbricated within a web of individual, social, and structural factors such as anxiety, depression, substance use, medical distrust, substandard housing, and poverty, along with historical and cultural influences – from the perspectives of the subpopulation of PLWH that faces the greatest challenges to sustained optimal ART adherence.

## Materials and methods

The present cross-sectional exploratory study drew on qualitative interview data with 18 adults living with HIV from African American/Black and Hispanic/Latino racial/ethnic and low socio-economic status backgrounds in New York City, NY, diverse with respect to the length of time living with HIV and experiences with ART. The primary aim of this exploratory study was to gather data on perspectives on HIV management including ART along with generating and critiquing potential future novel intervention efforts to support ART adherence. Although this exploratory study was not designed to investigate the construct of forgetting to take ART, we found during initial data analyses that forgetting was a useful organising concept, as we describe in more detail below. In study procedures and data analyses, we sought to reduce sources of bias. For example, the primary interviewer did not have contact with participants prior to the interview and participants were assured that their interviews would be kept confidential throughout the interviewing process. Further, the interview guide was comprised of open-ended questions, rather than direct questions, and instructions and questions were worded in a non-judgmental and accepting manner, to elicit participants’ perspectives on the phenomena under study without undue influence exerted from the research process.

## The local context

New York City is a location with a large HIV epidemic of approximately 127,000 PLWH, more than 75% of whom are African American/Black and Hispanic/Latino (New York City Department of Health and Mental Hygiene, 2019). HIV prevalence and HIV-related death rates are highly concentrated in the highest-poverty neighbourhoods of the city, which are predominantly African American/Black and Hispanic/Latino (New York City Department of Health and Mental Hygiene, 2019). The city provides a large network of HIV care settings (New York State Department of Health, 2016) and PLWH have access to HIV care and ART at low or no cost, regardless of immigration status (New York State Department of Health, 2016). New York City evidences serious racial/ethnic disparities in engagement along the HIV care continuum similar to national patterns, where African American/Black and Hispanic/Latino PLWH in low-SES locations show lower rates of engagement in HIV care and higher rates of suboptimal ART adherence compared to White PLWH in higher SES locations (LaVeist, 2005; Xia, Robbins, Lazar, Torian, & Braunstein, 2017).

### Recruitment

Participants were recruited using purposive sampling for a maximum variation on key domains. First, a modest number of participants were recruited from a Community Advisory Board in place for a separate ongoing study on people living with HIV conducted at our institution (*N* = 5), and these participants were invited to recruit their peers to enroll in the study. Recruitment efforts concentrated on enrolling participants with variability in length of time living with HIV and periods of optimal adherence/less-than-optimal adherence to ART. Recruitment continued until saturation was reached on core themes (Maxwell, 2012). The study was approved by the Institutional Review Board at New York University.

### Procedures

Participants contacted the study by telephone or were contacted by research staff and asked to participate in a 60–90-min in-depth semi-structured interview with a trained qualitative researcher. Participants gave signed informed consent before engaging in interviews. Interviews took place in a confidential location at a research study field site. Interviews were audio-recorded and professionally transcribed verbatim. Participants were offered compensation of $25 for their time as well as funds for roundtrip local transportation.

### Qualitative interview guide

The interview followed a semi-structured interview guide collectively developed by the research team and grounded in the theory of triadic influence described above. The guide was designed primarily to elicit participants’ past and present experiences with ART adherence. The guide included open-ended questions and prompts. Further, the guide focused on and/or elicited discussion of sensitive topics including HIV status, substance use, and incarceration. As noted above, instructions and questions were worded in a non-judgmental and accepting manner and participants were assured that their responses would be kept confidential. Further, participants were informed they could decline to answer any question without penalty.

Questions focused on individual-level domains such as the participant’s experiences with and perspectives on ART (e.g. What has it been like in the past for you to be on HIV medication? What is it like now?), motivation/readiness to take ART (Would you say you ‘wanted’ to take HIV medication? Did you feel ‘ready’ to take HIV medication?), and emotions about ART (How much do your feelings about HIV medications help or hinder your ability to take ART every day?). Social-level factors included relationships with providers (How can health care providers and health care settings help people take their HIV medication every day?) and stigma. Structural-level factors included factors such as stable housing (Some people have told us that their immediate needs today such as stable housing are more important to them than the idea of having to take ART today for good health in the future. Do you ever feel that way?). Throughout the interview process, the interview guide was updated to reflect newly emergent codes. Indeed, as noted above, the theme of forgetting to take ART was not emphasised in the first version of the semi-structured interview guide. However, we found it was a compelling and recurring construct early in the data collection process and was added to the guide. We also assessed a set of socio-demographic and background characteristics; namely, age, sex, race/ethnicity, number of years since HIV diagnosis, number and duration of times taking ART in the past, whether on ART in the past month, and the average level of adherence to ART on a 4-point Likert-type scale (not at all, fair, good/some of the time, very good/most of the time).

### Data analysis

The data analysis strategy was both theory-driven and inductive, and utilised a directed content analysis approach (Hseih & Shannon, 2005). Data analysis procedures were constructed to minimise bias. First, a primary researcher trained in anthropology analysed interview transcripts and developed an initial code list informed by the theory of triadic influence consisting of constructs related to optimal and suboptimal ART adherence, such as problems associated with low socioeconomic status, a desire for good health, motivation/readiness for ART, substance use, depression and anxiety, reasons for missed ART doses, including forgetting, and intentionally versus unintentionally missed doses. We also coded for factors related to history, culture, and race/ethnicity (e.g. the legacy of early zidovudine [AZT] monotherapy, discrimination, medical distrust, and counter-narratives about the origins of HIV). Next, a second trained qualitative researcher coded the interview transcripts and met frequently with the primary data analyst. Codes were further refined, and discrepancies were resolved by consensus (Braun & Clarke, 2013). Findings from this initial round of coding were then presented to the larger research team which formed an interpretive community (Wolf, 2003; Yardley, 2000) and codes were further refined. Then, codes were combined into larger themes in an iterative process and in collaboration with an interpretive community. The multi-level theoretical model enabled the research team to attend to the complex intersections of structural, social, and individual barriers to optimal ART adherence. As noted above, the theme of forgetting and its potential meanings and causes emerged organically throughout these qualitative interviews and served as the primary focus of the analysis for the present paper. The research team was made up of eight members who were men and women from White, Asian, and Latino/a backgrounds. The primary data analyst was trained as a medical anthropologist and was highly experienced with HIV research with diverse populations. Positionality challenges related to sex, gender, race/ethnicity, power, health, socioeconomic status, and privilege were intentionally addressed throughout the reflexive data collection process through reflection and training, which focused on how these types of issues might impact the interviewing process and data collection (Aronson, 2007). Throughout the study, for instance, we sought to address our own positionality by encouraging and fostering a continuing dialogue between staff and participants related to the aforementioned differences, as well as differences in HIV status and substance use history. Methodological rigour of the analysis was maintained through an audit trail of process and analytic memos and periodic debriefing with the larger research team, which included experts in HIV care and medication adherence issues (Osterberg & Blaschke, 2005).

## Results

Of the 18 participants interviewed, 56% were men and 44% were women. Most were African American/Black (79%), and the remainder were Hispanic/Latino. All were from low socio-economic status backgrounds and received public health insurance for low-income individuals (Medicaid). Participants ranged between the ages of 50 and 69 years and had been living with HIV from between 3 and 33 years, with an average of 21 years living with HIV. Most had participated in HIV-related support or therapy groups and substance use treatment programmes, although the duration of their involvement varied. All had taken ART for substantial periods in the past, along with episodes of ART discontinuation. At the time of the present study, 60% reported taking ART with optimal adherence in the past month. Although not included in the assessment of sociodemographic characteristics, we found substance use, past and/or current, was highly prevalent in this sample. During their interviews, most participants (78%) mentioned tobacco use or smoking, 61% discussed crack or powder cocaine, and alcohol, ‘dope’, heroin, injection drug use, and pills were also mentioned commonly in transcripts. A third of participants discussed past incarceration, jail, or prison, suggesting involvement in the criminal justice system was fairly common. Thus, participants exhibited a substantial number of serious risk factors that have the potential to interfere with optimal ART adherence. Names used below are pseudonyms and some identifying details have been changed to protect participants’ anonymity.

### Overview

Participants in the present study recounted their histories, generally very long histories, of managing HIV infection and ART adherence. Thus, results yielded a rich retrospective overview of the challenges inherent in maintaining optimal adherence to ART over decades of living with HIV, as well as successes and lessons learned. We found participants understood the importance of optimal ART adherence as their best chance for a long and healthy life. Moreover, they identified a number of factors that either promoted or impeded optimal ART adherence, some of which are fairly well-described in the literature to date, such as the importance of stable housing, as well several under-studied factors, as described below. Overall, participants highlighted the challenges inherent in taking medication for a chronic health condition that is both complex and stigmatised, particularly in the context of chronic poverty. Findings highlighted that the larger social and organisational structures in which African American/Black and Hispanic/Latino PLWH were embedded did not sufficiently support optimal ART adherence.

Consistent with existing epidemiological studies, optimal ART adherence, even once achieved, was not a static state or phenomenon, and participants described factors driving the dynamic nature of ART adherence patterns. These included the evolving nature of their perspectives on what it meant to be living with HIV. Further, life circumstances such as lost housing and changes in social/romantic relationships, which often triggered mental health and substance use problems, influenced ART adherence decisions and behaviours. Participants reported the ability to take ART optimally when using alcohol and/or illicit drugs socially or at non-hazardous levels. However, it was substantially more difficult for participants to prioritise ART in times of heavy or problematic substance use, although participants did often still consider missing or stopping ART a choice in this context. Nonetheless, those who missed ART doses, including in times of heavy substance use, generally re-grouped and started again. Moreover, participants described their complex and often shifting emotional responses to ART. Indeed, taking ART was rarely described as habitual, easy, or emotionally neutral. Participants viewed intrinsic motivation to take ART as fundamental to optimal ART adherence, but such motivation was both hard-won and commonly disrupted in times of hardship. Thus, ART adherence decisions and behaviours required constant recalibration. Missed ART doses commonly led to long periods of ART discontinuation, and it was often challenging for participants to harness internal and external resources to re-initiate ART and re-establish optimal adherence patterns. Overall, participants described the strategies they had developed to manage and sometimes redefine ART adherence, and the contextual and psychosocial factors that influenced those decisions and behaviours.

The concept of forgetting to take ART doses was prominent in participants’ narratives of managing ART adherence. However, as we describe below, forgetting to take one’s HIV medications was far more complex than a simple lapse of memory, and findings underscored the need for a closer examination of both what it means to be optimally adherent to ART and what it means to forget to take ART. Throughout the interviews, participants shifted between various explanations for the reasons they forgot to take ART doses. At times, forgetting was mentioned as occurring most frequently during times of high emotional stress and/or depression. At others, forgetting to take ART was closely associated with an ambivalent or even traumatic emotional response to both their HIV medications and the burden of HIV-related stigma. Moreover, forgetting to take ART frequently involved participants taking intentional or semi-intentional ‘holidays’ from their regimens. In still other cases, forgetting to take ART was associated with external circumstances that precluded taking medication doses altogether. Thus, we found forgetting to take ART emerged as a primary means of managing ART, and coping with living with HIV, over the long term. Importantly, participants highlighted that the line between true forgetting and intentionally missing doses was frequently blurred. Indeed, throughout the interviews, participants often either implicitly or explicitly declined a clear-cut distinction between accident and intent. Moreover, depending on the age of the participant, ambivalence about taking ART was often closely linked with direct memories of, or the historical legacy of, knowledge about AZT monotherapy in the early days of the epidemic, which participants described as experimental, being given in high doses, and causing serious side effects but with limited efficacy.

Health care providers were described as vital to participants’ abilities to manage HIV infection. However, participants experienced health care providers as a variable in how well they understood the complexities of managing HIV infection and optimal ART adherence. Participants commonly told their providers they forgot to take ART, but without disclosing or being asked to disclose the intentional, semi-intentional, or unintentional decisions or circumstances, whether internal or external, that contributed to those forgotten ART doses. Indeed, in the present study, participants repeatedly described forgetting as the reason for suboptimal ART adherence and only linked forgetting to its underlying causes (e.g. unstable housing, lack of structure, substance use, stress, stigma) through a process of extended reflection. Conversely, patient-provider relationships in which patients were encouraged to contribute to the consultation dialogue contributed to what participants viewed as more favourable HIV outcomes.

Study findings were grouped into six interrelated themes: (1) forgetting to take ART served as a shorthand description of a complex phenomenon, but was rarely a simple lapse of memory, and often involved intentional or semi-intentional breaks from ART; (2) forgetting was described as a means of handling the difficult emotional context within which HIV management and optimal ART adherence take place, including regarding the challenge of adaptation to the HIV diagnosis and HIV-related stigma; (3) adverse life events played a primary role in contributing to mental health distress and substance use problems, which in turn was related to disrupting optimal ART adherence, especially because living in poverty allowed for few buffering resources; (4) the legacy of AZT monotherapy and its relationship to present-day ART adherence, often grounded in trauma and loss, disrupted optimal adherence, including by contributing to medical distrust; (5) aspects of patient-provider interactions promoted or impeded the discussion of the factors underlying forgetting and optimal ART adherence; and (6) intrinsic motivation was critical for optimal ART adherence and as a buffer against forgetting. The findings focused mainly on the challenges inherent in sustained optimal ART adherence for this population living in the lowest socioeconomic strata, but also imply or describe strategies participants used to successfully sustain optimal ART adherence, along with factors that supported optimal ART adherence. Some themes address the issue of forgetting to take ART doses directly and others describe factors that make forgetting more or less likely. We describe each of these six themes below.

### Forgetting ART doses as a shorthand description

We found the phenomenon of forgetting to take ART was not a simple, unitary experience of failing to remember to or inadvertently neglecting to take a dose of ART. Participants commonly demonstrated an awareness, that appeared to be at least partially intentional and in the conscious mind, that forgetting to take ART was quite often more complicated than a simple lapse in memory. Throughout their interviews, participants oscillated between ascribing missed ART doses to simple forgetting and intentional decision-making. In many, if not most instances, forgetting reflected ambivalence about taking ART. For Sal, a Hispanic man in his mid-fifties who was diagnosed with HIV over 25 years ago, forgetting medication and the need to meet other priorities blended together. Recounting his history of heavy substance use, Sal explained the following:

And, to me, every time I was using [drugs], you know, if I had the choice or to wait – take your medication, get high, take your medication, get high, I would end up getting high, getting high and forgetting the medication if I could sell it, you know.

Indeed, selling ART to corrupt pharmacies was a common survival strategy in this sample, although doing so is illegal for pharmacies and places them at risk for criminal prosecution (Kuehn, 2014).

Fred, a Black man in his mid-forties, described the reasons for missed ART doses leading to ART discontinuation. As shown below, his explanation of his behaviour evolved from describing forgetting as the primary cause, to missed doses being related to the underlying issues of housing instability and ‘stress’.

The situation I was in I was just forgetting every day to take my meds. Every day, I would think about it after I get to work, or you know when I'm at work. Then when I get home, I'd forget. And I’d wake up and I’m like, damn, I forgot to take my meds. […] Then, when I’d think about it, I’d take it. […] But that wasn’t good because they say you’re supposed to take the meds at the same time every day. So, it was because my situation I was in, I was moving, and I was staying from place [to place]. I was staying with this person and that person, and I was in a shelter, then I was staying with somebody else. And then I was stressed, so I just stopped taking my meds all together. And I haven’t took my meds in, like, almost 30 days now.

Indeed, the process of forgetting was commonly experienced by participants as a fluid and shifting phenomenon, as was evidenced in the tendency for them to transition almost seamlessly between varying degrees of intentionality and understanding of their own behaviour.

Further, forgetting one’s medication also was often grounded in a set of complex and personal guidelines for what some participants referred to as ‘little vacations’. Participants commonly detailed reasons for intentionally taking regular breaks from their medication, which they referred to as ‘hiatuses’, ‘breaks’, and ‘holidays’. Beatrice, for instance, was a Black woman in her mid-forties who voluntarily participated in several programmes to support optimal ART adherence including Directly Observed Therapy (DOT) during most weekdays, and an intervention using a smartphone-based application that provided compensation for optimal adherence by self-report. Nevertheless, she often intentionally missed ART doses, particularly on the weekends, in order to avoid the side effects of what she referred to as ‘very toxic’ medication:

Well, you know, I mean, I stopped, I took it, I stopped, I took it, you know, but I was always in contact with the doctor when I went on those little holidays, you know, breaks. Two days here, three days there. You know, to overcome that side effect. […] I did the medication adherence [intervention] so they would text me at a specific time every day. There was times when sometimes I took it, sometimes, I clicked okay [on the adherence smartphone app] and I didn’t take it.

For Beatrice, intentionally missing ART doses and volunteering for DOT and other adherence-support interventions were not at odds. In fact, for Beatrice, knowing that she would not forget her ART medication on the days she was participating in the DOT programme provided the space within which she could prioritise her health in other ways.

Similarly, Jade, an African American woman in her mid-fifties who had been living with HIV for approximately 20 years, described taking an occasional ‘hiatus’ as her personal strategy for staying simultaneously healthy and at least moderately adherent to ART:

Something I have done since I have been on these meds and since I noticed the meds messed up with my body, I take one day off a month. Every month, I take one day off. The doctor told me, you should not do that. I said, listen, I think my body needs a rest for one day, just for one day. I do not think it is hurting me because I get right back on my regimen. That is what I do now is just take one day a month. Take that one day off and then I get right back on the pills.

After initially isolating herself for eight years because, as she noted, ‘I felt I had a big “A” on my chest and everybody knew about (my HIV status),’ Jade regarded these short breaks as a key part of her own current adherence and HIV management strategy.

### Forgetting as a means of managing the emotional context of HIV

Participants stressed that their perspectives on living with HIV and their feelings about ART and ART adherence were dynamic and influenced by both internal/psychosocial and external factors. Participants’ perspectives on ART were related in large measure to their initial reactions to receiving their HIV diagnosis and the difficulties they faced adapting to the new diagnosis. The process of adaptation to the HIV diagnosis was typically complicated by continuous and re-lived emotional trauma and a number of internalised stigmas. Indeed, we found stigma surrounding HIV remained a critical barrier to sustaining optimal adherence to ART. Harold, a Black man in his early fifties diagnosed with HIV while incarcerated about a decade years ago, described that he continued to struggle with both acceptance of his diagnosis as well as ART adherence, and attributed these struggles directly to HIV-related stigmas:

Yeah, I struggled [with my diagnosis]. That was the hardest part about it – accepting that I was going to have to die. Even though you know you’re eventually going to die but to have something accelerate it like HIV could – it was hard for me. I mean, I was thinking of all the possibilities I was losing as in having kids, having a meaningful relationship and so forth. So, I lost my purpose to live. That was my reason for struggling with the whole thing [taking ART without forgetting doses]. […] But [even] now I’m stuck with this stigma of either I’m gay or I’m strung out on crack or something like that, and I get treated as such. And that’s like stuff that I’m still dealing with now with the stigma of [HIV].

Although Harold understood the role of discrimination in his reluctance to accept his diagnosis and therefore to regularly take HIV medication without forgetting doses, feeling disrespected and marginalised nonetheless remained significant obstacles to optimal ART adherence. He went on to describe how he managed stigma and took ART consistently:

But I’m getting to the point now where I don’t care [about the stigma], I’m living with it. I’m not that stereotypical person [with HIV] and that’s what driving me to keep taking my medication. […] Because now a days if you’re not taking your medication, it’s something deep. You know what I’m saying? [It’s] stigma.

Samuel was an African American man in his early fifties who was diagnosed approximately 10 years ago, who took occasional breaks from his medication, firmly understood HIV as ‘man-made’, a common counter-narrative about HIV, and had struggled with disclosing his HIV status to his partner. He described experiencing denial after his initial diagnosis, related in part to HIV-related stigma, as follows:

It was just that [when diagnosed] I was in denial, too. Like, damn, how did this happen to me? You know what I’m saying? I was in disarray. I didn’t know what to take, should I trust this [medication], is it really gonna make me better? Or worse? … It’s a lot of [expletive], man. People are scared to admit up with what they have, or what they’re dealing with and that’s real. It is. And it’s all out here, you know what I’m saying? Either you own up [to] it, and that’s why a lot of people have passed for not owning up to it and taking their meds. See then a lot of people they’re like, oh damn, they’re gonna think I’m gay, or, you know what I’m saying?

Patricia, a 50-year-old Black woman diagnosed with HIV approximately 10 years ago, discussed how despite having accepted her HIV diagnosis, she continued to hide her medication from others and keep her HIV status ‘on the down low’ because, as she described, stigma associated with HIV affected her ability to take ART, which she viewed as a visible reminder of her HIV status both to herself and to others. Despite having accepted her diagnosis, optimal adherence remained challenging. She noted:

Back then [when I was diagnosed] it was like you got the monster. How did you get it? You are nasty. You are going to die and hurt people. They are going to throw something at you. That is the whole thing about this disease. You do not know which way to go with it [in terms of disclosure]. […] Now, I have fatigue. Runny diarrhoea sometimes, night sweats, and I get dizzy sometimes. That is not as bad as other people that is living with it, so I am grateful. I try not to complain. Sometimes, I forget my meds because I just forget. Now, I have a pill bottle [which reduces forgetting]. […] [But], I forget. There are other things on my plate.

Similarly, for Beatrice, introduced above, anxieties related to disclosing her HIV status, whether intentionally or unintentionally, remained a direct, concrete barrier to ART adherence at times:

I’m a little ashamed to take my medication in front of people I have not disclosed to … . And I’m not gonna take my medication sitting in a restaurant. Like, I’m just not gonna do it, you know? I’ll sit in the restaurant because there’s other people sitting there eating, but I’m not gonna pull out my medication and take it.

Relatedly, when reflecting on the experience of living with HIV and HIV stigma more generally, Fred, introduced above, linked his desire to live, and therefore to take his ART regularly, directly to the stigma surrounding HIV:

Because yeah, after a while [living with HIV stigma] you lose your purpose to live, like what is the purpose of living? If you don’t find it within yourself because you have to have some kind of human contact, a hug, a kiss or what have you, what have you, and a lot of people they don’t have that for the simple fact they living with this HIV. Because people got disowned by their family. Even though that’s not in all cases, I’m just painting the whole bush.

For these and other participants, despite having sometimes overcome their initial reluctance to accept their HIV diagnosis, and despite having developed innovative strategies to deal with HIV-related stigma, the emotional contexts within which HIV and ART are situated nonetheless continued to significantly impede consistent use of ART and contributed to missed or forgotten doses. In most cases, the primary barrier to ART adherence was HIV stigma, and ART use was a signal to oneself and possibly others that the individual was living with HIV, thus raising the possibility of being stigmatised.

### Life events contribute to mental health distress and disrupt optimal ART adherence

Participants pointed out that difficulties adhering to ART often stemmed from immediate and pressing emotional and material factors. They noted that forgetting to take one’s HIV medication frequently occurred during bouts of depression related to acute life stresses, which also commonly coincided with periods of increased substance use. Lapses in adherence were commonly associated with emotional distress related to such concerns as paying rent and other bills, caring for children or grandchildren, managing health-related obligations such as doctor’s appointments, and experiencing relationship problems. Patricia, introduced above, described how everyday pressures in her life related to forgotten ART doses:

Right now, I never heard nobody talking about not taking their meds [intentionally], but other stuff going on like the kids, the grandkids, the rent. [They have issues like] they want to work, they cannot [work], their health. It takes a toll. When you keep taking medication, it takes a toll. The doctors want to give you this, give you that, give you this. Take this. There are side effects … Some days, we, as humans, forget.

Thus, Patricia’s experience serves as a reminder that daily adherence is challenging for all individuals, and that persons living with HIV commonly experience a wide range of issues that ‘take a toll’. Similarly, despite generally feeling positive about taking HIV medications, Terence, an African American man in his early fifties diagnosed with HIV approximately 20 years ago, nevertheless described being unable to see himself taking ART indefinitely, and situated regularly forgetting to take his medication within the context of a ‘screw-it attitude’ resulting from chronic stress and depression:

So, it was because my situation I was in, I was moving and I was staying from place to [place] – I was staying with this person and that person, and I was in a shelter, then I was staying with somebody else. And then I was stressed, so I just stopped taking my meds all together. And I haven’t took my meds in, like, almost 30 days now … No, it was never a conscious decision. No, I wouldn’t just say no I’m not gonna take it. I just kept forgetting every day … Yeah, so this is like, for me taking my medication, if I’m not stressed and I’m not doing a lot, I can take my meds, you know. It’s just that, ‘cause right now, I’m just, I’m not happy. I’m just not happy with my living situation, I’m not happy with the – not even the job situation. I’m just not happy, like, with the environment, you know, of people that I’m around right now.

Relatedly, for many participants forgetting to take ART doses had to do with a ‘frame of mind’ most frequently described as depression. Lawrence, an African American man in his early fifties who was diagnosed with HIV over 30 years ago, situated his tendency to forget his medication within the context of the material and emotional difficulties of ‘getting back on the right track’:

You forget [to take an ART dose] because you’re rushing and things of that nature. Or a person may have emotional problems. They may get depressed over anything. Oh, [expletive], it’s raining. Get depressed. And, you know, and through the depression, they don’t take the med … You know, I got two jobs, I got two kids who’re living with me all of a sudden. So, you know, I’m busy with them, I forget. But, you know, I can’t double up on the meds, so then I have to skip one and I have to wait ‘til the next day. And, you know, I’ve been doing that lately. That’s been going on with me pretty much in a nutshell.

Sal, introduced above, had what he now described as a heavily ritualised relationship with ART adherence that nonetheless included occasionally ‘breaking the cycle’:

It’s really bad if you start forgetting to take your medication or you get into an argument or you’re going through a bad day and you say, ah, I’m not gonna take it. Or you’re gonna turn around and have a fight with your girlfriend and you’re gonna go and go pick up and use drugs. Some people can’t just do it by themselves, you know.

Thus, participants identified critical relationships among acute life stressors (i.e. ‘having other stuff on your plate’), depression and other mental health issues, and substance use, all of which in turn contributed to forgetting one’s medication, whether intentionally, semi-intentionally, or unintentionally, which, in turn, regularly interfered with achieving their overall goal of good health. For these participants, while ART adherence and overall good health were valued and prioritised in a general sense, forgetting to take or choosing to miss an occasional dose of ART was nonetheless viewed as oftentimes necessary for medical, social, and psychological reasons.

### Legacy of early AZT monotherapy, trauma, and loss

Most participants in the present study had been living with HIV for more than two decades. As a result, forgetting ART or intentionally skipping ART doses was often part of a self-fashioned adherence strategy that focused on long-term health. Further, ART adherence patterns and decisions commonly took place within the context of both lived experiences with AZT monotherapy, and long-standing cultural narratives regarding AZT monotherapy’s toxicity. Throughout these interviews, many participants recalled witnessing friends, family, and community members deteriorate physically and die due to AZT monotherapy, while still others explicitly attributed the fact that they were still alive to *not* taking AZT monotherapy when it was available. Indeed, achieving optimal adherence, however defined, often meant navigating a complex landscape partly composed of medical distrust, past losses and trauma, and ongoing continuous traumatic stress, as well as fears regarding long-term side effects of ART such as organ damage and neuropathy, largely influenced by the scientific community’s first efforts at controlling HIV beginning in the late 1980s; namely, AZT monotherapy. Emmett was a 55-year-old Black man who was diagnosed with HIV 36 years ago, and who described a lack of readiness to initiate ART until 18 years after his diagnosis. He spoke of ‘skipping around’ and ‘going on little vacations’ from ART and explained that this is in part because he understands that ‘the AZT is still in the meds’, albeit in smaller quantities. As he explained:

Because [when I was first diagnosed] I was afraid. I lost a lot of associates, a lot of friends. I was feeling, you know – that’s the thing. This is funny, but people of colour, we have nappy hair. Then you see them again and it’s all straight, you know there’s a problem there. And then sometimes then you see the skin tone looks ashen grey – ashen grey. I don’t want to look like that. And then you could see – change in the face. You know the loss of weight in the face. That was one of those [side effects]. I wasn’t vain, but then people were dying from [AZT].

The effects of AZT monotherapy’s legacy were similar for Jared, a Black man in his early fifties who learned he was living with HIV approximately 30 years ago. While he proudly stated that he had consistently achieved undetectable HIV viral load levels for approximately 10 years, Jared remembered struggling to trust HIV medications after having experienced the negative effects of AZT:

Off and on I was like basically non-compliant for a long time because I’m from back in the dinosaur days when there was like terrible side effects and I wasn’t adherent for a long time. […] I only became adherent when I was placed on [a newer ART regimen]. I just wasn’t educated, didn’t know and there was just so much stuff [so many different regimens]. And being sick wasn’t part of my daily – I couldn’t do it. […] I began to become adherent, and my viral load started to come down and my CD4 went up.

Notably, for Jared, part of his own strategy for achieving an undetectable HIV viral load included making a clear distinction between ‘adherence’ and ‘compliance’, with the former being far more in line with his own ideas about how to manage his health than with his health care provider’s views. Indeed, the term ‘compliance’ highlights acting in accordance with a directive from another, while ‘adherence’ is defined as persistence or steady maintenance, suggesting the individual’s agency in medication-taking behaviour (Friedland, 1997).

Other participants described intense personal adverse experiences with AZT, which had left them with varying degrees of distrust of even the most recent HIV medications, which they feared were potentially toxic. Lennard, a Black man in his late fifties who was diagnosed with HIV while incarcerated over 25 years ago, and who was subsequently prescribed AZT monotherapy, described his experience as follows:

And it’s the weirdest thing, that particular substance. I felt it spread out through my system all the way out to every pore and capillary. I was aware of that over a two-day, three-day period. It’s like when your shoes are a size and a half too small, there’s no such thing of not being aware of it on a constant. After two days of that, that was it … They gave me a week supply, but I did two days of it and I said, ‘to hell with that.’

Although he now has what he described as an improved relationship with ART, he has recently been ‘on and off with the medication’, explaining that ‘I’ve got other stuff that impacts my [health] – I have asthma that impacts my life much worse than the HIV.’

Similarly, Lawrence, a Black man in his early fifties who was diagnosed with HIV while incarcerated approximately 30 years ago, noted that after having felt heavily pressured to take AZT while in prison, the following occurred:

What happened was one of my partners that was doing life took the med and flushed it down the toilet. Yeah, he took the medicine from me. He’s, like, what the [expletive] are you doing? You’re killing yourself. Because my skin was turning grey. I was getting these [lines] –it’s still there. You see that? You see that black line right there? That’s AZT. That’s what it does to your nails. So, you know. And I was actually losing more weight. I was 120 pounds. No, I actually-no, I’m lying. 117 [pounds]. I’m six feet tall. Skin and bones. Picture my face sunk completely in. So, when my people started seeing that, it was like, oh man, [expletive] this. They took my [expletive], they went through my locker. They found all the meds, they threw it in the garbage, and they nursed me back to health.

Not only did Lawrence express gratitude for the life-saving actions of his fellow inmates, but upon being released from prison Lawrence began ‘preaching against people taking [AZT]’, stating that:

So that’s what my thinking was with these [expletive] meds, they’re trying to kill us all … I still believe that the government used the gay population as a means to put out this virus to control the population because it’s mostly affected Latinos and Blacks.

Nita, a Latinx woman diagnosed almost 30 years ago and who recalled the ‘dramatic’ adverse effects of AZT monotherapy on her son, also remembered a time during which adhering to HIV medications was particularly burdensome for her and noted that:

But the thing is I just didn’t forget to take my medication. […] There’s forgetting where people want to forget. I didn’t want to go to that medication stuff when I first started, 15 different kinds of pills. That was toxic, you know.

Finally, it is important to note that even for individuals with more recent HIV diagnoses, AZT-related ambivalence and generalised medical distrust were still often important factors when thinking about ART initiation and optimal adherence, and that contributed to forgetting ART doses. Val, a 51-year-old Black woman who was diagnosed with HIV in 2006, recalled an initial distrust of both the reality of her HIV diagnosis and the medications prescribed, stating that, ‘Back then, who knows what they do with the medication back then.’ Within this context, AZT-related fears and distrust continued to occasionally resurface for many, playing a role in forgetting ART doses, whether intentional or semi-intentional, among those living with HIV. This was true for those who had been prescribed AZT monotherapy, as well as later recipients of information on the fraught, earliest days of HIV treatment.

### Aspects of patient-provider interactions promoted or impeded ART adherence

Our analysis demonstrated that patient-provider relationship elements were closely linked with African American/Black and Hispanic/Latino PLWH’s care satisfaction as well as their motivation to take ART with optimal adherence. Most participants had experiences with various providers over their HIV careers, some engaging in practices they found promoted optimal ART adherence and others that did not. We focus in this section on the approaches or stances adopted by providers that participants described as promoting open dialogue and productive patient-provider relationships. First, participants typically referenced the harm-reduction approach as respectful, acceptable, and helpful; that is, a plan of action to aid an individual in minimising harms corresponding to particular at-risk behaviours. When applied in the context of substance use, harm-reduction recognises the patient’s ongoing degree of drug use and develops strategies focused on decreasing adverse outcomes associated with use. Participants also highlighted that patient-provider relationships established within a framework of collaboration and acceptance in an unconditional manner led to the creation of optimal settings for discussing ART adherence, disclosing forgetting, and unpacking the factors that underlie forgetting.

In particular, participants commonly highlighted the importance of feeling safe enough in the patient-provider relationship to be honest about their substance use, stating that a non-judgmental approach to substance use, and similarly regarding ART adherence and forgetting doses, positively affected their inclination to sustain HIV treatment. Jared (mentioned above) recounted how being allowed to freely delve into his thoughts and feelings with what he referred to as ‘running and ripping’ in regard to his at-risk lifestyle correlated with his gradually improved adherence:

When I first started taking the Truvada, Reyataz, and Norvir [ART regimens], I got to the point where I would at least, if I missed the dose, I would at least go back and take it later in the day. But if I was smoking crack that morning it wasn’t going to happen. But I would go back [and take ART] later in the day. So, I kinda got acclimated to missing less doses, you know. [My doctor] was part of that. ‘Cause I didn’t never divulge my substance use to my doctors before him. And he was like, Jared, I know you’re using, you know, and – I trusted him, you know. And he was like this is how this is gonna work and this is how – and he made me – I say made me, but he convinced me to be honest with him. And by being honest with him, I got better with the medicine.

At a later point in the interview, Jared continued to describe how he frequently did not think about taking his medicine, especially when he was actively using crack cocaine. However, in being comfortable enough to discuss his substance use, his physician encouraged him to develop a routine that involved remembering to take his pills while preparing to go to bed whenever Jared returned home from one of his ‘outings’.

In a similar narrative, Tyler (mentioned above) shared how his physician opted to use a harm-reduction strategy rather than insisting that he completely refrain from substance use:

When I first was diagnosed … I remember her name; my first physician. And when we had this discussion about the medications and my substance abuse, she says, I’m not so worried about you with the cocaine and crack cocaine. What I would encourage you to do is in the same kind of routine that you use your street drugs, you take these HIV meds. So, it was like a piggyback kind of routine … And it worked, because I became adherent. So, over the course of, I guess years, I just became more adherent to taking the meds and I would slowly wean myself off of the other street drugs, you know.

In addition to his experience of interconnectivity between positive patient-provider dynamics and HIV medication, Tyler discussed how matters ‘clicked’ following his physician’s advice to think of taking his HIV medication using a similar routine as his street drugs:

By her not stigmatizing a label – because I’ve had other physicians that they’re very judgmental. You know, you’re doing drugs, you’re not going to take these meds, you’re going to sell them, so I’m not going to give you these medications. […] They figured this is just going to make you worse. But she was very proactive of her telling me, well, I just would like you to [use] the same routine that you do [for] your street drugs, I would like you to take these HIV meds [the same way]. […] So, that kind of clicked in and that made me adherent.

Lennard, introduced above, is a participant who had been on and off his ART regimens in part due to the lasting negative effects of his first experiences with AZT and resultant medical distrust. Lennard commended his physician for acknowledging that absolute adherence cannot be considered a realistic scenario for all patients to experience:

You miss doses here and there. But then one doctor was brave enough to say basically if you’re regular enough about it you’re pretty pickled [that is, you are receiving a high dose of ART even if you occasionally miss doses]. So that’s not supposed do so much damage missing a dose here and there. And that’s reality. Anyone who says much else besides from citing serious circumstances for huge blocks of missed doses is lying.

Indeed, for the majority of participants, independent attempts to improve ART adherence seemed to occur when an environment of refuge was created by the physician. It was a common theme that normalising a patient’s struggle, as well as the absence of criticism and judgment towards at-risk behaviours, catalysed the patient to improve or re-commit to optimal adherence. Often times, promoting adaptation of street drug routines to HIV treatment could be seen as serving as a type of memory tool to organically integrate ART into the participant’s daily events, habits, and routines. Further, providers’ non-judgmental and accepting attitudes allowed participants to analyse and address the factors underlying forgotten or otherwise missed ART doses.

### The importance of intrinsic motivation in optimal ART adherence

As noted above, participants uncovered and described factors that interfered with optimal ART adherence, including life stressors, depression, substance use, the fear of ART toxicity, medical distrust, and HIV-related stigma, as well as one factor that promoted optimal ART adherence; namely, open and honest patient-provider relationships. Moreover, participants noted that feeling intrinsically motivated to take ART was a necessary first step in developing an adherence strategy, and high levels of intrinsic motivation to take ART could help buffer against challenges to good adherence and also reduce forgetting. For instance, participants such as Sal (mentioned above) frequently repeated the idiom that ‘you can lead a horse to water, but you can’t make it drink’, and stressed the importance of intrinsic motivation for optimal adherence to ART:

It’s like you can take the horse to the water but you can’t force him to drink, you know. And I was already determined to just get that help [to take ART], you know. And, once I started getting the help and I seen, you know, doors opening up for me, I just started like, you know, boom, let me see what this is about, let me see what this is about, and, you know, gave me motivation and stuff.

For many, overcoming barriers to optimal adherence such as substance use, stigma-based concerns regarding being seen taking HIV medication, or depression need to be grounded in a genuinely internal desire to maintain good health. Nita (mentioned above), for instance, saw the utility of external incentives to take ART, such as financial compensation programmes provided in some community-based organisations and HIV care settings, but noted the following:

But it’s up to you to stand up on your own two feet and do it [take ART]. […] It has to come from the person, themselves, you know.

Similarly, Maria (mentioned above) expressed a common sentiment amongst people who are currently optimally adherent to ART:

I could give you a bottle of medication, but if you don’t want to take it, you know, it’s up to you not to take it. But, if you want something – if you wanna be healthier and live a little longer, you could take your medication. It’s up to you if you wanna live, you know. I don’t understand why people sell their medication when it’s something that’s helping you.

Once again, however, it must be noted that being intrinsically motivated to become or to remain optimally adherent to ART was not always synonymous with being compliant. Indeed, several participants in this study distinguished clearly between adherence and compliance, with the former being more autonomously shaped than the latter, which was more often used to describe the degree to which participants followed advice from their health care providers.

### Summary of results

In nearly all instances noted above, forgetting to take one’s ART medication, intentionally or semi-intentionally missing doses, describing one’s inability to take ART, or configuring a personal definition of ‘good adherence’ all emerged in part as socially acceptable responses to both internally and externally directed questions regarding why one was not optimally adherent to ART. That is, within the context of perceived pressure from health care providers, friends, family, or even oneself, forgetting to take ART frequently sidestepped a more or less conscious intention to miss doses or to redefine what it means to be optimally adherent to ART, and in this sense might also be seen as a relatively passive form of an individual’s assertion of autonomy. Moreover, in many cases forgetting or intentionally missing ART doses allowed participants to focus on other life priorities. That is, whether or not directly attributed by participants to stress, substance use, depression, or inattention, forgetting to take ART carved out a space within which participants could attend to other aspects of their lives.

## Discussion

Adherence to medications is a universal challenge for individuals across the spectrum of chronic health conditions (Tobias, 2009), and has been the ‘Achilles’ heel’ of HIV treatment since the first HIV ART agent was introduced in the 1980s (Gadkari & McHorney, 2012). The present exploratory qualitative study sought to revisit the question of ART adherence, in the context of the third decade of the HIV epidemic and from the perspectives of the sub-populations that face the greatest challenges to optimal adherence and subsequent HIV viral suppression. Although we did not initially seek to investigate the topic of forgetting to take ART in the semi-structured interview guide used in the present study, the concept emerged as an important and multifaceted theme during data collection. The qualitative nature of this study, therefore, allowed us to explore experiences of and factors underlying the phenomenon of forgetting to take ART when it emerged organically throughout the interviews. Although forgetting as a barrier to optimal ART adherence has been well documented in the empirical literature (Barfod et al., 2006; Chesney et al., 2000; Marecek, Fine, & Kidder, 2001; Sauceda et al., 2018), it has rarely been explored qualitatively. Thus, the present study makes an important contribution to the literature by focusing in-depth on this critical aspect of optimal ART adherence.

Study findings indicate that many participants are aware, on some level, of the degree to which forgetting to take ART doses may provide an opportunity to focus on other priorities, whether in regard to their health or other domains. Some past research has focused on the concept of ‘intentional non-adherence’ (Kalichman et al., 2013). Results from the present study, however, do not suggest that intentional non-adherence is the only mechanism at play. Instead, we found forgetting is sometimes intentional, and that the lines between intentional and non-intentional missed ART doses are often blurred, in part as a means of managing one’s ART adherence in a manner deemed optimal for that individual, and other times a function of external circumstances. Moreover, at times participants could not articulate the reasons for forgetting to take ART doses. Therefore, in the discussion that follows, we intend the term ‘forgetting to take ART doses’ to include the continuum of expressed motives that underlie missing ART doses, ranging from actual unintentional forgetting, semi-intentional missed doses, to intentionally missing doses as an active HIV management strategy, along with the inability to take ART doses because of internal or external factors and missed doses for unknown reasons.

Consistent with previous research, we found forgetting to take one’s ART medication is one of the most frequently self-reported barriers to optimal ART adherence. When explored in-depth with participants, however, it became evident that forgetting medication is rarely a unitary or isolated phenomenon. Rather, forgetting to take ART doses is deeply entwined with other risk factors prevalent in this population at the lowest socio-economic strata such as stress, anxiety, depression, negative or aversive emotions related to living with HIV, HIV-related stigma, substance use problems, medical distrust, unstable housing, adverse life events, trauma, as well as an overall desire for wellbeing. Thus, results highlight the challenges inherent in managing a lifelong, difficult, and stigmatised health condition, particularly in the context of arduous life circumstances such as poverty and unstable housing. Rather than being a simple lapse in memory, we found among this population of African American/Black and Hispanic/Latino PLWH, forgetting to take ART doses is a complex and layered phenomenon that often coincides or overlaps with intentionally missed doses, which in turn is oftentimes part of an individual’s larger strategy of long-term management of ART. Thus, the statement that one forgot to take ART is in many cases a type of shorthand for a wide range of decisions and behaviours, along with internal and external factors, that drive adherence behaviour. Further, in comparison to other reasons for missing ART doses, forgetting is relatively socially acceptable and easily understood by others. Although studies have shown that prospective memory, that is, remembering to carry out a planned action in the future, plays an important role in medication adherence (Fassin & Rechtman, 2009), the current study’s findings indicate that even when individuals plan to take their medication at a later time, unfavourable social and structural conditions such as HIV stigma, extreme poverty, and unstable housing, and mitigating factors such as substance use, anxiety, depression, or interruptions to daily routines, often lead to missed ART doses. As noted above, African American/Black and Hispanic/Latino individuals are over-represented in the population of PLWH and are also less likely to exhibit optimal ART adherence compared to White PLWH (Beer et al., 2016; Samji et al., 2013). Yet, African American/Black and Hispanic/Latino PLWH are also more likely than White PLWH to experience the unfavourable structural, social, and individual-level factors identified in the present study that serve as impediments to optimal ART adherence (Pellowski, Kalichman, Matthews, & Adler, 2013). Thus, understanding forgetting to take ART is necessary for addressing persistent racial/ethnic disparities in HIV-related behaviour and health outcomes.

We found forgetting to take ART doses can be a means of managing HIV and ART over the long term. Indeed, forgetting sometimes occurs alongside the explicitly stated desire to *not* take medication. In fact, most frequently the primary difference between intentionally missed doses of ART and true forgetting to take ART doses seemed to be the level of remorse participants express. Moreover, for most participants in the present study, neither forgetting one’s medication nor intentionally missing doses necessarily conflict with participants’ perceptions of the need to sustain optimal ART adherence. Rather, true forgetting and/or missing doses, whether intentionally or semi-intentionally, appear to be overlapping modes of managing optimal ART adherence. We found that forgetting is often closely associated with the fear or experience of unwanted ART side effects, complicated by medical distrust, as well as with concerns regarding ART’s potential to cause long term organ damage and other issues related to an individual’s physical health. Indeed, intentionally missing doses, or taking a brief hiatus from one’s HIV medication, seems to serve as a way for individuals to make healthy adjustments to what they understand to be potentially harmful pharmaceutical regimens. This is also evidenced in the way that, regardless of the level of intentionality, many participants set relatively explicit rules for missing doses (e.g. not more than one or two missed doses per month, no more than two missed days in a row, etc.). Thus, forgetting is one strategy participants use to manage their medication in the way they deem optimal. While optimal adherence is generally defined as taking ‘every dose, every day’, participants may have discovered that they can forget to take or otherwise miss ART doses and still achieve HIV viral suppression (Byrd et al., 2019). Yet, the present study suggests that strategically missing ART doses is not yet socially acceptable. Thus, participants generally describe missed doses as forgetting to take ART. This may be the most socially acceptable way to describe personal ART management strategies, even if not a precise or accurate description of why ART doses are missed.

The difficulties inherent in discussing missed ART doses with health care providers play a primary role in patients reporting they forgot to take ART. In particular, the fact that health care encounters are relatively short may perpetuate this shorthand description of missed doses, because providers do not have the time to explore what the patient means by forgetting to take ART, and/or may not be aware that they could do so or that there would be a benefit to doing so. Moreover, the present study suggests that social service and health care providers who do not sufficiently recognise the psychosocial and structural barriers to optimal ART adherence might actually prove to be an additional obstacle to PLWH reflecting on the factors that underlie their adherence patterns. Yet because health care providers are so pivotal in helping patients achieve and sustain optimal adherence, the fact that there is little time to explore what it means to forget to take ART is a missed opportunity to support patients.

But if forgetting is complicated, then so is remembering to take ART, which in many cases might require deprioritising other parts of one’s life. For some participants, reminders to take ART are more useful when provided within the context of genuine care and concern. For participants whose efforts at ART routinisation were described as successful, this involved not only the utilisation of daily reminders, but also of broader life changes, and most were most frequently related to improved patient-provider relationships. Thus, participants in the present study identify a number of characteristics of health care providers that foster good communication, allowing them to go beyond a simple description of forgetting to take ART, to the factors, beliefs, and decisions that underlie suboptimal adherence patterns.

The literature suggests numerous approaches developed and/or taken up by health care providers to support their patients in disclosing aspects of HIV management generally considered socially undesirable. Jaiswal, Griffin-Tomas, Singer, and Lekas (2018) found that African American/Black and Hispanic/Latino PLWH inconsistently engaged in HIV care valued patient-centred health care in which they felt genuinely heard and cared for by their HIV clinicians (Jaiswal et al., 2018). They conclude patient-centred medicine can be particularly useful for PLWH who experience social and economic marginalisation and who are inconsistently engaged or adherent to ART at suboptimal levels (Jaiswal et al., 2018). Further, the motivational interviewing (MI) approach integrated into HIV care has the potential to improve the quality of patient-provider relationships including frank discussion of socially undesirable behaviours (Flickinger et al., 2013). MI is a client-centred, non-judgmental, and directive counselling style that promotes behaviour change by supporting autonomy, exploring ambivalence about behaviour change, and eliciting the client’s own motivation to change (Miller & Rollnick, 1991). Yet, HIV care providers are not routinely taught the MI approach (Flickinger et al., 2013). Beach et al. (2015) trained HIV care providers in communication skills grounded in MI and found increased dialogue about ART adherence among providers and patients.

We found the desire for good health is universal in this sample. Every participant interviewed stated a desire to be healthy, with ART being seen as at least somewhat necessary to reach the goal of good health, regardless of whether he/she had been highly adherent to ART in the past, had good adherence at the time of the interview or thought that he/she might be highly adherent in the future. Throughout, when discussing forgetting their ART doses, participants’ statements indicate they are comfortable holding a set of contradictory cognitive positions regarding good health in general, and ART in particular. Namely, participants’ emotional experiences when contemplating ART, as well as with other medications and health behaviours, are located at the complex intersection of both an internalised, socially desirable goal of ‘good health’, and an emotionally, socially, and structurally fraught perspective on HIV, HIV care, and ART.

Consistent with other research findings (Saberi et al., 2015), this study’s results suggest that while external memory aids such as phone call reminders, alarms, and smartphone apps might assist individuals with optimal ART adherence to some degree, these are likely to be insufficient outside of interventions focused on structural barriers to adherence, mental health care, substance use treatment, and motivation and readiness for ART. For participants in this study, the significant difficulties presented by structural inequalities not only serve as immediate material barriers to optimal ART adherence, such as the need to sell HIV medication to meet financial needs, but also either create or exacerbate anxiety and depression. Forgetting also frequently takes place within the context of medical distrust and a sense of the loss of autonomy associated with a needing to take a life-long pharmacological regimen. Similarly, results from this study indicate that taking ART is also often experienced as a return to a traumatic past, including the historical context within which an individual suspected that he/she contracted HIV, as well as the trauma of the initial diagnosis (Rothbauer, 2008). As a result, many participants describe feeling the need to ‘take a break’ from HIV medication, which is frequently described as forgetting ART doses. In this sense, forgetting medication or intentionally missing ART doses might be viewed as part of a larger mental health management strategy as well.

Similar to past studies, these results highlight the importance of understanding individuals’ conceptualisations of what it means to be optimally adherent to ART as both flexible and individually defined (Engler, Lènàrt, Lessard, Toupin, & Lebouché, 2018). For participants in this study, perspectives on adherence (e.g. how often to miss doses, how long to take breaks) vary not only between participants, but also throughout individuals’ own HIV management trajectories. In fact, for many participants, alternative or idiosyncratic conceptualisations of optimal adherence frequently take precedence over those recommended by even the most trusted health care providers, and participants often share their views on optimal adherence with providers. Indeed, for many participants in this study, rather than being viewed as a barrier to adherence, forgetting one’s medication may be viewed instead as an individualised, and at times even integral, part of living with HIV. In Table 1 we present a range of practical recommendations for health care settings that emerged from the present study.

**Table 1.** Practical recommendations for HIV care and service settings that emerged from the present study.

Overall lesson learned	Specific recommendations
Forgetting is a socially acceptable shorthand description of a complex phenomenon	Health care and social service providers can be sensitised to the fact that ‘forgetting’ is an expectable aspect of managing ART over the long term, because patients use ‘forgetting’ as a way to manage ART and living with HIV Providers can help patients unpack the precise meaning of and factors underlying forgetting (e.g. true forgetting, forgetting due to ambivalence about ART, semi-intentional missed doses, intentional missed doses, life context prevented ART doses being taken, the patient does not entirely know why doses were missed, etc.) Providers can understand that factors driving forgetting may not be obvious to PLWH; in other cases, forgetting may be an intentional or semi-intentional means of HIV management PLWH may experience shame or other negative emotions associated with forgetting, but providers can cultivate a non-judgmental environment by accepting forgetting as one expectable aspect of managing ART over the long-term
Optimal adherence to ART is rarely natural, easy, or permanent	In the context of highly efficacious and tolerable ART regimens, even sophisticated and well-intentioned providers may become desensitised to how challenging maintaining optimal ART adherence can be for many patients Assumptions in clinical settings that optimal ART adherence will necessarily be sustained can be challenged, to reduce socially desirable responding from patients and to foster open discussion of barriers to ART adherence and reasons for forgetting Policies and practices in clinical settings can and should support the range of psychosocial and structural factors that underlie optimal adherence to thereby reduce ‘forgetting’ (e.g. stable housing, harm reduction)
PLWH’s perspectives on living with HIV and taking ART play a role in forgetting	Acceptance of an HIV diagnosis can take time and difficulties coming to terms with the reality of the diagnosis may contribute to forgetting; providers can query patients about their experience of living with HIV Counter-narratives about HIV and its treatments interfere with motivation to adhere to ART, but there are few places where patients can discuss counter-narratives. Providers can introduce the topic of counter-narratives (e.g. HIV was man-made to exterminate populations of colour, there is a cure for HIV that is being withheld, ART is toxic) and explore as appropriate but without attempting to change patients’ minds about the counter-narrative. Negative emotions about living with HIV and taking ART, including fear and distrust of ART, may be potent and contribute to forgetting. Distrust and fear of ART are typically more salient among African American/Black and Hispanic/Latino populations compared to White populations, grounded in historical events and present-day structural racism
Trauma is endemic and contributes to forgetting	Trauma is endemic among those living in poverty, and HIV infection itself is often experienced as trauma Taking ART is an emotional reminder that one is living with HIV infection and these emotions can be challenging to tolerate, particularly when practical or psychosocial stability is disrupted Trauma-informed care approaches can augment existing care models
Intrinsic motivation for ART is fundamental, but may be overlooked	Providers may assume PLHW have sufficient intrinsic motivation to take ART when prescribing, but it may not be; low levels of intrinsic motivation for ART can contribute to forgetting Intrinsic motivation is necessary but not sufficient; habits along with social and structural supports can prevent or reduce forgetting
Certain life conditions make missed ART doses less likely	A range of life circumstances and psychosocial social conditions make forgetting less likely including: stable housing, a strong social support network, acceptance of HIV status, emotional readiness for ART, and good management of mental health and/or substance use concerns PLWH from low socio-economic status backgrounds are commonly unemployed, because of health or in order to retain safety net benefits. Yet, employment creates structure and routine, which can reduce forgetting. Community-based and AIDS service organisations provide vital structure, but more roles for PLWH are needed to provide structure and generativity (e.g. peer mentorship programmes)
HIV care providers may benefit from training to elicit the underlying causes of forgetting	Short health care encounters impede providers’ abilities to understand what their patients mean by forgetting to take ART Patient-centred medical care can improve clinical encounters, including assisting patients with understanding what is meant by forgetting to take ART and the factors that underlie forgetting Approaches such as motivational interviewing show promise in clinical care encounters to engage patients and guide them toward optimal personal health care decisions Social workers and other service providers who interface with patients can provide services that complement medical care, and elicit the various meanings of forgetting to take ART

### Limitations

The present study has several limitations. One general limitation is the purposive sampling method, which may limit its generalisability to the population of African American/Black and Hispanic/Latino PLWH as a whole. Yet purposive sampling is consistent with the goals of qualitative research, which aims for depth rather than breadth. A second limitation has to do with the small sample size. Nonetheless, saturation was reached on core themes. Moreover, the small sample size did not allow us to examine sex or racial/ethnic differences in detail, a gap that future studies on this topic can address. Last, the present study did not include respondent triangulation, such as interviews with health care providers or other stakeholders. Indeed, such triangulation would have allowed us to examine forgetting from different perspectives and thereby validate results through cross verification (Rothbauer, 2008).

## Conclusions

Although the most common reason PLWH give for missing ART doses is that they forgot to take it, forgetting to take ART is not generally a simple lapse in memory. Instead, forgetting to take ART is a shorthand description of a multi-faceted phenomenon. In some cases, forgetting is intentional, as a personal strategy for managing ART (e.g. taking a break to manage ART toxicity), and in others, forgetting arises from ambivalence about ART, or may be semi-intentional. We also found that participants at times cannot describe why they forgot to take ART. Thus, in many cases, it appears forgetting lies somewhere between intentional and non-intentional processes. In other cases, PLWH may be unable to take ART. Moreover, a range of factors make forgetting, whether intentional, not intentional, or somewhere in-between, more or less likely. For example, stable housing and social support can buffer challenges to adherence and reduce the chances of forgetting, while depression, extreme poverty, stigma, and substance use at hazardous levels increase the chances of forgetting. Strong patient-provider relationships can help patients move beyond a description of simple forgetting as the main reason for missed ART doses, to the underlying meaning of forgetting, to help PLWH articulate their approaches to ART adherence and identify behavioural practices they can put in place to maximise optimal ART adherence and health.

## Ethics approval and consent to participate

This study was carried out in accordance with the recommendations of the University Committee on Activities Involving Human Subjects (UCAIHS) at New York University (FWA#00006386). The protocol was approved by the Institutional Review Board at New York University. All subjects gave written informed consent for study activities in accordance with the Declaration of Helsinki.

## Data Availability

The datasets used and/or analysed during the current study are available from the corresponding author on reasonable request.
